# Opportunities and challenges of targeting c-Met in the treatment of digestive tumors

**DOI:** 10.3389/fonc.2022.923260

**Published:** 2022-08-01

**Authors:** Zhengchao Zhang, Dong Li, Heng Yun, Jie Tong, Wei Liu, Keqiang Chai, Tongwei Zeng, Zhenghua Gao, Yongqiang Xie

**Affiliations:** ^1^ Department of General Surgery, The Third Affiliated Hospital of Gansu University of Traditional Chinese Medicine, Baiyin, China; ^2^ Department of General Surgery, Second Hospital of Lanzhou University, Lanzhou, China

**Keywords:** c-Met, digestive system tumors, gastric cancer, hepatocellular carcinoma, pancreatic cancer, colorectal cancer, targeted therapy, adoptive immunotherapy

## Abstract

At present, a large number of studies have demonstrated that c-Met generally exerts a crucial function of promoting tumor cells proliferation and differentiation in digestive system tumors. c-Met also mediates tumor progression and drug resistance by signaling interactions with other oncogenic molecules and then activating downstream pathways. Therefore, c-Met is a promising target for the treatment of digestive system tumors. Many anti-tumor therapies targeting c-Met (tyrosine kinase inhibitors, monoclonal antibodies, and adoptive immunotherapy) have been developed in treating digestive system tumors. Some drugs have been successfully applied to clinic, but most of them are defective due to their efficacy and complications. In order to promote the clinical application of targeting c-Met drugs in digestive system tumors, it is necessary to further explore the mechanism of c-Met action in digestive system tumors and optimize the anti-tumor treatment of targeting c-Met drugs. Through reading a large number of literatures, the author systematically reviewed the biological functions and molecular mechanisms of c-Met associated with tumor and summarized the current status of targeting c-Met in the treatment of digestive system tumors so as to provide new ideas for the treatment of digestive system tumors.

## Introduction

Digestive system tumors (DSTs) mainly include gastric cancer(GC), hepatocellular carcinoma (HCC), pancreatic cancer(PC) and colorectal cancer(CRC), which are general cancers in worldwide (1). Although surgery, chemotherapy and molecular targeted therapy have been widely used in the treatment of DSTs, the prognosis of most advanced DSTs patients is still poor (2). Therefore, it is crucial to explore effective therapeutic targets and strategies for middle-advanced DSTs. Several studies have shown that c-Met is a promising treating target for DSTs (3–5). In recent years, clinical trials of treating DSTs based on c-Met targets have achieved favourable security, and c-Met inhibitors have been found to be an efficient treatment regimen in combination with other drugs (6).

MET gene is involved not only in the proliferation, differentiation and invasion of various human tumor cells, but also in the resistance of anti-tumor drugs (7). It is overexpressed in many human tumors, including respiratory system tumors, DSTs (8–11), urinary system tumors (12, 13) and reproductive system tumors (14, 15). Previous researches have demonstrated that inhibition of c-Met signaling, such as non-small cell lung cancer (NSCLC), HCC, GC, PC, CRC, ovarian cancer, bladder cancer (16–25), is an efficient anti-tumor strategy for many tumors. c-Met has also been found to participate in the drug resistance of epidermal growth factor receptor-tyrosine kinase inhibitors (EGFR-TKIs) treating NSCLC patients (25). The combination of c-Met inhibitors and EGFR-TKIs might be considered a promising treatment option.

Researches have demonstrated that c-Met normally mediates downstream signals by combining with its corresponding ligand, hepatocyte growth factor (HGF), to promote the proliferation and differentiation of tumor cells. It was also found that c-Met can also activate downstream pathways through signaling interaction with some carcinogenic molecules in the absence of ligands (26, 27). Therefore, the mechanism of c-Met promoting tumor proliferation and drug resistance is complicated. In order to promote the clinical application of c-Met targeted drugs for DSTs, it is necessary to explore the mechanism of c-Met action in DSTs so as to optimize the anti-tumor activity and treatment regimens of c-Met drugs. This paper systematically reviews the biological functions of c-Met related to tumor formation and development, and summarizes the researches on targeting c-Met in the treatment of DSTs, in order to bring new ideas of targeting c-Met in the treatment of DSTs.

## The structure of c-Met/HGF and its tumor-related signaling pathway

**Figure 1:**
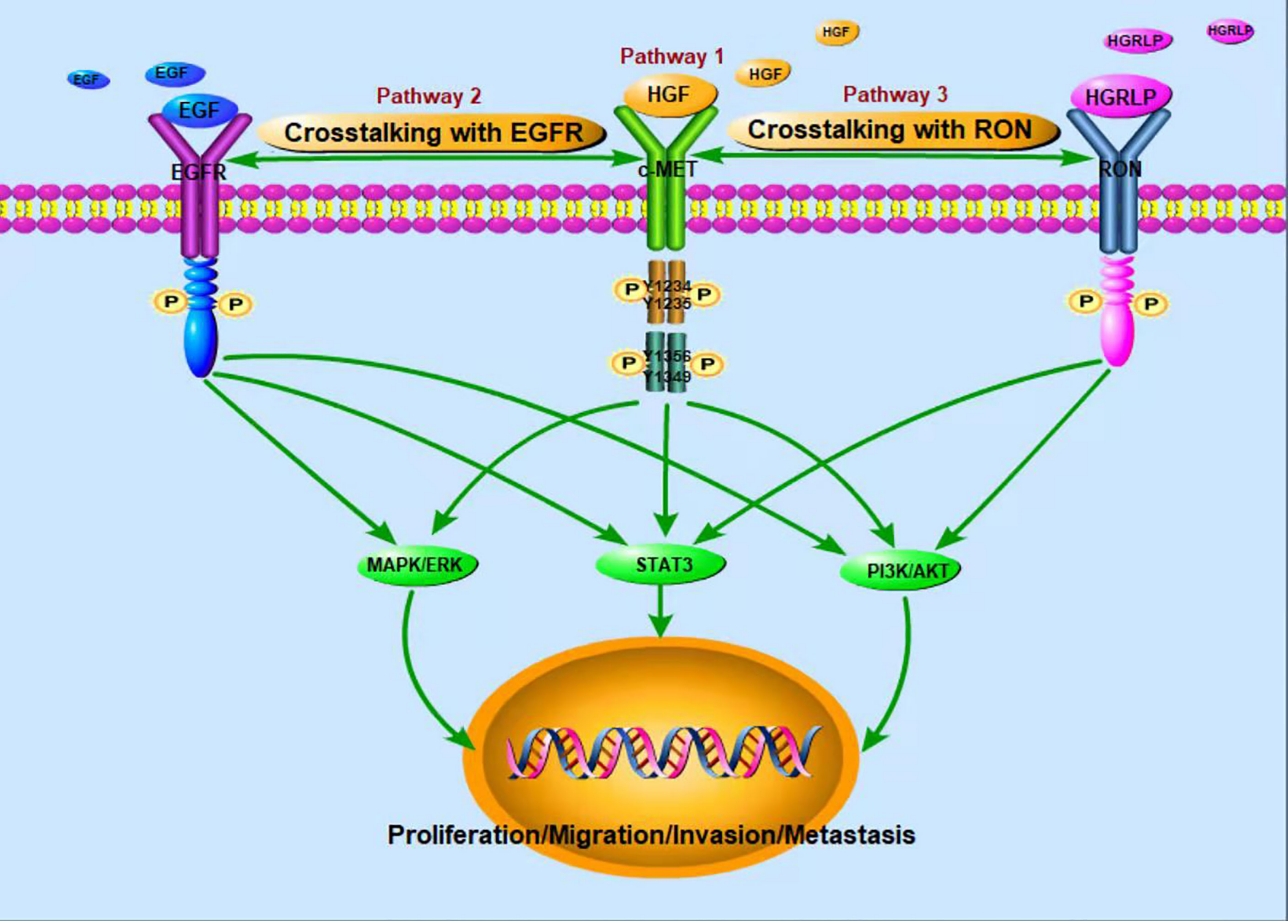
Activation of c-Met tumor-related signaling pathways.

c-Met is a glycosylated membrane protein consisting of transmembrane β chains (145 kDa) and extracellular α chains (50 kDa). HGF, as a ligand of c-Met, is composed of a 103 kDa soluble heterodimer, which consists of α-chain and β-chain linked together by disulfide bonds (28). Binding of HGF and c-Met leads to autophosphorylation of tyrosine residues Y1234 and Y1235 in the tyrosine kinase domain, which further activates autophosphorylation of the Y1349 and Y1356 tyrosine domains near the COOH (**Figure 1**). For example, the adaptive molecule GRB2-associated-binding protein 1 (29) is a scaffold protein connector containing a c-Met binding site, which provides a binding site for effectors containing the SrC-Homology-2 domain. The same way as translational protein SH2, phosphoinositol 3 kinase, protein tyrosine phosphatase with SH2 domain, phospholipase Cc1, signal transduction and transcription activator 3, etc. Therefore, the binding of HGF and c-Met regulates cellular biological functions by activating the above-mentioned downstream signaling pathways (30–34). The HGF/c-Met pathway is also regulated by other proteins, such as integrin, which promotes the activation of RAS, PI3K, plexinB1, signaling hormone and death receptor Fas (35). Through these second messengers, c-Met downstream signaling pathways trigger many biological activities, such as cell proliferation, cell survival, motor function and morphological changes (36, 37).

In recent years, studies have found that EGFR and c-Met are often co-expressed in tumors, and they share a common downstream signaling pathway, such as ERK, MAPK, PI3K, AKT pathways. The interaction between EGFR and c-Met has been reported in HCC (38). Besides, Yoshiaki Nakamura et al. (39)found that cetuximab induced MET gene mutation and amplification in advanced GC, suggesting that EGFR-targeted therapy may mediate drug resistance through MET amplification and/or proteinhyperactivation. Further studies showed that the signal interaction between c-Met and EGFR mediated anti-tumor resistance (40, 41). Yuying Zhu et al. (27) also found that up-regulated c-Met signal can be used as a compensation mechanism for weakening EGFR family signal so as to maintain the proliferation of chemotherapy resistant breast tumor cells. The combination of c-Met and EGFR targeted therapy was also found to synergistically inhibit proliferation of drug-resistant cells *in vitro* and *in vivo*. According to the above findings, simultaneous targeting of these two targets may be promising in the treatment of tumors. A number of clinical studies have also verified the antitumor activity of targeted EGFR and c-Met combination therapy (42–44). Therefore, in order to perfect the anti-tumor activity of c-Met targeted therapy and develop its function in treating drug-resistant tumors, future studies should further explore the interaction mechanism between c-Met and EGFR.

In addition, RON (Recepteur d ‘Origine Nantais) receptor is a member of the tyrosine kinase receptor family. The homologous similarity between the extracellular domain and kinase domain and c-Met domain was 25% and 63%, respectively. The activation of RON can lead to the activation of MAPK, PI3K and other signaling pathways. RON ligand is hepatocyte growth factor-like protein (HGFLP) (45). According to the sequence homology with HGF, the cDNA encoding HGFLP was isolated for the first time and named HGFLP (46). HGFLP is mainly secreted by liver cells and is a single chain precursor with no biological activity. RON and c-Met, interactive activation between which has been demonstrated, are co-expressed in many tumors; what’s more, recent studies have suggested that c-Met trans-activation of RON may be a hallmark of cancer cells (26). Both c-Met and RON play important functional roles in embryonic development and organogenesis, and are overexpressed or abnormally activated in various tumors (47). S Zhao et al. (48) found that RON knockout can increase the intensity and duration of c-Met signal, suggesting that c-Met signal could compensate for the loss of RON signal. Therefore, RON and c-Met exert a crucial role in tumor genesis and development through their dynamic complementarity.

## Tumor-related biological functions and regulation of c-Met/HGF

c-Met is essential for several events including angiogenesis, myoblast migration, bone remodeling, and nerve germination during the processes of embryogenesis (49). In vertebrate adulthood, c-Met has been found to be constitutively expressed in epithelial cells during liver regeneration and wound healing (50). c-Met/HGF kinase pathway is inactivated in normal tissues, but activated in various tumor tissues (51). As a proto-oncogene, MET mediates tumor cell proliferation, invasion, angiogenesis, chemotherapy resistance, epithelial-mesenchymal transformation (52–55).

HGF is defined as a secretory factor responsible for enhancing cell motility, invasion and causing cell dispersal (56). HGF in tumor microenvironment can be derived from both tumor cells and tumor associated stromal cells (56). Binding of actived HGF and c-Met leads to oligomerization of receptors, activation, autophosphorylation of tyrosine residues, and substrate docking, thereby activating downstream signaling processes (36, 57). Activation of other tyrosine kinases is also found to be involved in enhancing the HGF/c-Met signaling pathway. EGFR plays a critical role in promoting c-Met mediated cell proliferation, cell invasion, and cell survival (58). EGFR activation can induce SRC-dependent c-Met activation, which is ligand-independent (59). Similarly, at the downstream of c-Met activation, the release of PGE2 induced by COX2 increases the activity of matrix metalloproteinases and releases EGFR ligands, such as bidirectional regulatory proteins (60). Therefore, EGFR and c-Met promote tumor progression through signal interaction. Other carcinogenic mechanisms have also been found to enhance the effects of c-Met. c-Met interacts with insulin-like growth factor 1 receptors could promote tumor cell invasion and migration (61). In addition, hypoxia positively regulates c-Met activity through tumor angiogenesis (62). These findings suggest a complex interaction system that regulates and controls the size and duration of c-Met signaling.

The association of c-Met activity with tumor growth, invasion, and poor prognosis has been demonstrated (35). C-Met aberrations occur in approximately 50% of HCC patients and can be caused by gene mutations, gene amplification, increased mRNA expression, and receptor overexpression (63). Although c-Met has a potentially beneficial role in chronic liver disease, increased activity may initiate or promote the development and progression of HCC. Therefore, c-Met is considered to be an important factor in the regulation of liver disease and a carcinogen driver of HCC. In PC, increasing of MET transcription leads to c-Met overexpression, and c-Met increasing promotes tumor genesis and development through a variety of mechanisms (64). C-Met expression is 5-7 times higher in PC tissues than in adjacent tissues, providing a large therapeutic safety window for targeted c-Met therapy in PC (65). In addition, inappropriate activation of c-Met pathway in GC was found to be mainly due to the amplification and mutation of Met gene leading to c-Met protein overexpression (66). About 4-10% of gastric cancer patients have MET amplification, and 50% of advanced gastric cancer patients have C-MET protein overexpression (67, 68). High expression of c-Met was also detected in CRC and has been observed to be associated with tumor invasion, lymph node and liver metastasis. Therefore, c-Met plays an important role in the diagnosis and treatment of DSTs.

## Application of c-Met inhibitors in DSTs

In the past few years, a number of c-Met inhibitors have been developed for therapeutic studies in tumors and have shown significant antitumor activity in preclinical studies of DSTs (69–73). Some c-Met inhibitors have been used in the clinical treatment of DSTs and achieved certain efficacy. In addition, several new c-Met inhibitors and drug therapy strategies are being developed for DSTs (**Table 1**). Therefore, it is of great importance to deeply understand the regulatory mechanism of HGF/c-Met pathway in finding effective c-Met inhibitors and therapeutic strategies for DSTs.

**Table 1:** Clinical studies on c-Met inhibitors in the treatment of DSTs.

Conditions	Interventions	First posted	Number enrolled	Phase	NCT number	State	Status
Gastric Cancer	Drug: crizotinib	May 6, 2015	2	Phase 2	NCT02435108	Korea	Completed
Advanced Solid Tumors	Drug:Capmatinib	October 5, 2016	36	Phase 1	NCT02925104	United States	Completed
Solid Tumors	Drug: Capmatinib	March 29, 2011	131	Phase 1	NCT01324479	United States	Completed
Gastric Cancer	Drug: MCLA-129	May 3, 2021	150	Phase 1 Phase 2	NCT04868877	United States	Recruiting
Solid Tumors	Genetic: SAIT301	November 20, 2014	16	Phase 1	NCT02296879	Korea	Completed
Solid Tumor, Adult	Drug: HLX55	November 19, 2019	98	Phase 1	NCT04169178	Taipei, Taiwan	Recruiting
Gastric Cancer Gastroesophageal Junction Adenocarcinoma	Drug: APL-101 Oral Capsules	June 5, 2017	201	Phase 1 Phase 2	NCT03175224	United States	Recruiting
Solid Tumors	Drug: tepotinib	April 16, 2013	12	Phase 1	NCT01832506	Japan	Completed
Advanced Solid Tumors Which Are cMET-dependent	Drug: capmatinib Drug: Nazartinib Drug: Gefitinib	February 2, 2017	40	Phase 2	NCT03040973	United States	Recruiting
Solid Tumor	Drug: SPH 3348	October 21, 2021	36	Phase 1	NCT05088070	China	Recruiting
Pancreatic Adenocarcinoma Metastatic	Drug: Cabozantinib Drug: Erlotinib	July 11, 2017	7	Phase 2	NCT03213626	United States	Terminated
Colorectal Cancer	Drug: MCLA-129	June 18, 2021	400	Phase 1 Phase 2	NCT04930432	China	Recruiting
Solid Tumors	Drug: tepotinib	November 17, 2009	149	Phase 1	NCT01014936	United States	Completed
Solid Cancers	Drug: bevacizumab •Drug: MetMAb	February 17, 2010	44	Phase 1	NCT01068977		Completed
Colorectal Cancer	Drug: PF-02341066 Drug: PD-0325901 Drug: Binimetinib	July 28, 2015	82	Phase 1	NCT02510001	United Kingdom	Completed
Solid Tumor	Drug: RC108	November 5, 2020	32	Phase 1	NCT04617314	China	Recruiting
Solid Tumors	Drug: HS-10241	May 3, 2016	7	Phase 1	NCT02759640	Australia	Completed
Solid Tumor	Drug: Tepotinib	December 1, 2020	100	Phase 2	NCT04647838	Korea	Recruiting
Hepatocellular Carcinoma	Drug: Tepotinib	April 16, 2014	66	Phase 1 Phase 2	NCT02115373	Germany	Completed
Liver Cancer	Drug: TIVANTINIB	January 7, 2014	386	Phase 3	NCT02029157	Japan	Completed
Advanced Solid Tumor	Drug: CAPMATINIB	March 7, 2012	44	Phase 1	NCT01546428	Japan	Completed
Solid Tumor	Drug: HS-10241	July 20, 2020	30	Phase 1	NCT04477057	China	Recruiting
Advanced Solid Tumors	Drug: AMG 337	December 3, 2010	111	Phase 1	NCT01253707	United States	Completed
Carcinoma, Hepatocellular	Drug: Foretinib	June 15, 2009	45	Phase 1	NCT00920192	Taiwan	Completed
Advanced Solid Tumors	Drug: ABT-700	November 16, 2011	74	Phase 1	NCT01472016		Completed

All clinicaltrials can be downloaded from www.clinicaltrials.gov (accessed February 28, 2022).

### Hepatocellular carcinoma

In 2020, hepatocellular carcinoma (HCC) was the sixth most common cancer and the third leading cause of cancer deaths in worldwide, with approximately 906,000 new cases and 830,000 deaths (74). Conventional chemotherapy has limited efficacy, and the 5-year survival rate of advanced HCC is less than 10% (75). Therefore, it is urgent to develop effective treatments for advanced HCC. Researchers studied the expression of c-Met in 62 HCC patients and its correlation with prognosis, they found that the 5-year survival rate of HCC patients with high expression of c-Met was significantly lower than that with low expression of c-Met (76). The study also found that high c-Met expression in HCC was associated with an increased incidence of intrahepatic metastasis. The study suggests that c-Met plays an important role in the formation and development of HCC and may be a promising therapeutic target. In recent years, c-Met inhibitors, multikinase inhibitors and some new drugs have been used in the treatment of HCC.

Tivantinib, a selective small molecule inhibitor of c-Met, has shown significant antitumor activity in phase I clinical trials for the treatment of HCC. However, hematologic toxicity of tivantinib, mainly with neutropenia, was observed in phase II clinical trials (77). Santoro et al. conducted a study in which 97 patients with advanced HCC were randomized to tivantinib or placebo. The median time of progression was longer in the tivantinib group compared with the placebo one. 10 patients with neutropenia and 8 with anemia in the tivantinib group was also found (78). However, a recent randomized, double-blind Phase III clinical study conducted by Rimassa et al. showed that tivantinib did not improve the overall survival of c-Met positive patients with advanced HCC (79). These studies indicate that tivantinib may have some limitations in the treatment of HCC.

Sorafenib, as a multikinase inhibitor, is currently the only confirmed first-line treatment for advanced HCC (80). Sorafenib obviously perfected overall survival in patients with advanced HCC; however, its antitumor activity was generally hampered by the development of drug resistance (81). Since then, researchers have identified c-Met high-selective inhibitors in combination with Sorafenib as a potential therapeutic strategy for HCC. JIANG X et al. (82) found that a new c-Met inhibitor (DE605) combined with Sorafenib could effectively induce HCC cells apoptosis *in vitro* and inhibit HCC metastasis *in vivo*. MinKe He et al. (83) conducted a randomized clinical trial of Sorafenib combined with oxaliplatin, fluorouracil and calcium folate in the treatment of HCC patients with portal vein invasion. The results showed that Sorafenib combined with chemotherapy significantly improved overall survival and had an acceptable toxic effect compared with single Sorafenib treatment. Therefore, Sorafenib combination therapy may be a good strategy for the treatment of HCC.

Tepotinib, which has been shown to inhibit the progression of c-Met positive HCC *in vivo*, is a highly selective c-Met inhibitor*in vivo (* 84). Baek-yeol Ryoo et al. (85) conducted a phase II clinical study (NCT01988493) to evaluate the role of Tepotinib in the treatment of advanced HCC. Patients treated with tepotinib had better progression-free survival and objective response rate than that with Sorafenib, and tepotinib showed better drug tolerance. Faced with the problem of Sorafenib resistance, Thomas Decaens et al. (86) conducted a phase II clinical study to evaluate the efficacy of Tepotinib in Sorafenib resistant patients with advanced HCC (NCT02115373). Tepotinib was generally well tolerated and showed good efficacy. These studies suggest that tepotinib may be more effective than Sorafenib in the treatment of advanced HCC, and tepotinib is effective in patients with Sorafenib resistance. Tepotinib has great potential in treating advanced HCC.

SU11274, a small molecule inhibitor of c-Met, could also inhibit the growth of HCC cells by inhibiting the activation of c-Met (87). Other c-Met inhibitors such as cabozantinib, Capmatinib, Golvatinib and foretiinib have also been reported in HCC treatment (88–90). All of these c-Met inhibitors have shown antitumor activity in preclinical studies of HCC. However, the efficacy and safety of these drugs need to be tested in future clinical studies. The peptide LZ8, as a c-Met inhibitor, is extracted from ganoderma lucidum and has anti-tumor activity in breast cancer, lung cancer, cervical cancer and HCC (91–94). As a Chinese herbal ingredient, the peptide LZ8 has been certified as safe, but clinical trials are still needed to verify its safety and effectiveness in HCC patients. Although c-Met inhibitors have great potential in the treatment of HCC, more clinical studies are needed to optimize the treatment regimen and improve the prognosis of patients with advanced HCC.

### Pancreatic cancer

Pancreatic cancer(PC) is a highly lethal disease with a 5-year survival rate of only 10% (95). The disease is often insidious, and most patients could not often be diagnosed until at advanced stage. In recent decades, with the advancement of diagnostic, treatment methods and techniques, the prognosis of PC patients has been significantly improved (96). However, its efficacy is still limited for advanced patients, and better treatment strategies need to be developed to solve the current situation. A large number of studies have confirmed that c-Met is involved in the formation and development of PC (91, 92, 97). Renzo et al. found that c-Met was up-regulated in most PDAC, while the expression level was very low in normal pancreatic tissues (97). In addition, other studies have shown that the c-Met/HGF pathway plays a crucial role in the progression, invasion, metastasis and therapeutic drug resistance of PC (48, 93). Therefore, c-Met may be a promising therapeutic target for PC.

Chenwei Li et al. (94) evaluated the tumorigenicity of PC cells with high expression of c-Met in NOD SCID mice, the results showed that cells with high expression of c-Met were more likely to develop tumor than those with negative expression of c-Met. Studies *in vitro* also showed that c-Met inhibitor XL184 could significantly suppress the formation of tumor globules, suggesting that cells with high expression c-Met increased the tumorigenic potential of mice. In NOD SCID mice, the use of c-Met inhibitors slowed tumor growth in pancreatic tumors. In addition, other studies have found that c-Met inhibitors PHA665752 and AMG102 can not only block the HGF/c-Met axis by reducing the phosphorylation level of c-Met, but also weaken the epithelial mesenchymal transformation and chemotherapy resistance (98). Firuzi et al. (99) also found that pancreatic stellate cells increased resistance to gemcitabine through the c-Met/HGF signaling pathway. Besides, Zhihong Xu et al. (100)found in preclinical studies that c-Met inhibitors combined with chemotherapy drugs could completely eliminate metastasis and significantly reduce tumor growth *in vivo*. Therefore, these studies suggest that c-Met inhibitors can not only inhibit the growth of c-Met expressing PC, but also serve as an important therapeutic option for patients with chemotherapy-resistant PC.

Furthermore, the sequence of c-Met expression in PC cells changed after irradiation, and the expression of c-Met was induced by irradiation instantaneously (101). Irradiation also enhanced downstream phosphorylated MET expression in a mouse model of *in vivo* subcutaneous tumors. Compared with cells with low expression of c-Met, PC cells with enhanced expression of c-Met after radiation had a higher malignant potential, including invasion and migration. Capmatinib has been shown to reverse this enhanced malignant potential by inhibiting c-Met expression. These studies not only explain the possible mechanism of PC progression after radiotherapy, but also provide a theoretical basis for radiotherapy combined with c-Met inhibitor therapy for PC.

Soichi Takiguchi et al. (102) evaluated the effect of Crizotinib on peritoneal spread of PC *in vivo*, the study found that Crizotinib reduced tumor burden and ascites accumulation. So crizotinib may be an effective drug in treating PC patients with peritoneal metastasis. In addition, Enliang Li et al. (103) found that c-Met inhibitors combined with PD-1/PD-L1 inhibitors could achieve better efficacy against PC *in situ* and subcutaneous mouse models, indicating that combination of c-Met and PD-1/PD-L1 inhibitors may be a charming choice for PC treatment.

### Gastric cancer

Gastric cancer (GC), causing more than 1 million new cases and an estimated 769,000 deaths in 2020, ranking respectively fifth and fourth globally in morbidity and mortality, remains an important cancer worldwide (74). Clinically, the prognosis of patients with advanced GC is still poor (104). Surgical resection, radiotherapy and chemotherapy for advanced GC patients have been widely used in clinical practice, but the efficacy is limited. Therefore, it is necessary to further explore the molecular mechanism of GC in order to find effective therapeutic targets. Researchers conducted Northern blot analysis, reverse transcription polymerase chain reaction and immunohistochemical staining on 45 patients with GC, and found that the expression of MET mRNA in GC tissues was 2 times and 7 times higher than that in normal adjacent tissues (105). c-Met was detected overexpression in 32 of all patients (71.1%), and was significantly overexpressed in GC tissue compared to normal ones. What’s more GC patients with high c-Met expression have a poor overall prognosis (106, 107). Therefore, c-Met is a potential therapeutic target for GC.

Haiyan Liao et al. (108) found that Volitinib inhibited downstream PI3K/Akt and MAPK signaling pathways by selectively inhibiting c-Met phosphorylation, and significantly inhibited proliferation of MKN45 cell lines with high c-Met expression *in vitro* and *in vivo*. Paul R. Gavine et al. (109) also found that volitinib could lead to significantly GC tumor cell growth stagnation *in vitro* experiments, but its anti-tumor activity was negligible in xenograft tumor model. The above *in vivo* results*in vivo* may be related to different tumor models, such as MKN45-derived CDX model used by Haiyan Liao, and PDX model used by Paul R. Gavine. After all, there are certain differences in target expression between CDX model and PDX model. Therefore, the efficacy of Volitinib in GC needs to be verified by more PDX models or organoids with high c-Met expression.

Tivantinib and SAR125844 are also widely studied as selective c-Met inhibitors. Bum Jun Kim et al. (110) evaluated the inhibitory effect of tivantinib on proliferation and migration of GC cells, and discussed the mechanism of tivantinib through carcinogenic pathway analysis. Oncogenic pathway analysis showed that tivantinib inhibited the expression of VEGF signal in GC cells in addition to the c-Met signaling pathway. Studies have shown that tivantinib has anti-tumor effect not only on GC cells with high expression of c-Met, but also on ones with expression of non-c-Met. Tivantinib has been studied in clinical trials in several different tumors, including NSCLC, HCC and metastatic GC. In a multicenter Phase II trial, 31 Japanese and Korean patients with metastatic GC were enrolled, 11 of whom had disease control (111). However, adverse events remain serious. SHITARA K et al. conducted a phase I clinical trial of SAR125844 treating GC. It involved 22 Asian patients with GC and showed moderate anti-tumor function in 2 of them, although adverse events were also common in these patients (112). Similar to the HCC treatment, most c-Met inhibitors still have Insufficient benefits for cancer treatment due to adverse events and limitations of c-Met positivity.

Tepotinib is an oral, potent, highly selective c-Met inhibitor. Sung-hwa Sohn et al. (113) evaluated the antitumor activity of Tepotinib in GC cell lines. Tepotinib showed good antitumor growth activity *in vitro* and in mouse models, with reduced levels of phosphorylated c-Met protein. The results of this study suggest that Tepotinib inhibits tumor growth and migration by negatively regulating c-Met induction. Kohei Shitara et al. (114) conducted a multi-center Phase I clinical trial (NCT01832506) to evaluate the dose tolerated by patients with solid tumors. Tepotinib was generally well tolerated, no dose-limiting toxicity was observed, and treatment-related adverse events were mainly in grade 1-2 GC patients. These studies suggest that Tepotinib may play a greater role in the treatment of GC due to its good tolerability.

Yashiro et al. (115) studied the clinical efficacy of SU11274 combined with irinotecan in the treatment of GC, and found that the effect of inhibiting tumor growth *in vivo* through combined administration was superior to that of any single drug therapy. SU11274 was found to inhibit the c-Met signaling pathway, thereby reducing the expression of uridine 50-bisphosphate-glucuronyltransferase 1A1 (UGT1A1), which is associated with irinotecan resistance. Since then, KRC-408, KRC-00715 and Simm530 have been identified as selective inhibitors of c-Met, all of which have been shown in preclinical studies to inhibit the growth of GC cells (69, 116–118). Still, more clinical studies are needed to verify its efficacy and safety.

### Colorectal cancer (CRC)

Colorectal cancer (CRC) is the third most common malignancy in the world and a familiar cause of cancer-related death. It is estimated that there were more than 1.9 million new CRC cases and 935,000 deaths of CRC in the United States in 2020, accounting for about one in 10 cancer cases and deaths (74). In recent decades, great progress has been made in CRC early diagnosis measures, combination chemotherapy, targeted drugs and surgical treatment, but there are still limitations in efficacy. Existing targeted drugs have poor efficacy on some CRC subtypes (119), and further research on the molecular mechanism of tumor genesis is needed to optimize the efficacy of targeted drug. Studies have shown that the high expression of c-Met in CRC is associated with tumor invasion and liver metastasis (120). In recent years, a large number of studies have devoted to figure out the role of HGF-MET signaling pathway in CRC, and illuminated that this pathway is a therapeutic target. Liu et al. (121)observed that the expression of c-Met in CRC mucosal tissues was significantly higher than that in normal ones. Takeuchi et al. (122) used real-time quantitative polymerase chain reaction to study 36 patients with early CRC, and found that the expression of MET in CRC tissues was significantly higher than that in normal colon mucosal tissues, and the high expression of c-Met was related to the depth of intestinal wall invasion and regional lymph node metastasis. Therefore, c-Met inhibitors are widely used in the studies of CRC treating.

Crizotinib, Capmatinib and Tivantinib are also used as selective inhibitors of c-Met in the treatment of CRC. Feng Du et al. (123) found that crizotinib could block the HGF/STAT3/SOX13/c-Met axis and significantly inhibit SOX13-mediated CRC migration, invasion and metastasis. Kyle C. Cuneo et al. (124) evaluated the radiosensitization of crizotinib in cetuximab resistant CRC cell lines. The results showed that crizotinib effectively increased the sensitivity of cetuximab KRAS mutant CRC cell lines to radiotherapy. Besides, Jean-pierre Delord et al. (6) conducted a clinical study of Capmatinib combined with Cetuximab in the treatment of c-Met positive metastatic CRC (NCT02205398). 13 patients were found to have no dose-limiting toxicity and tumors shrank 29-44% in 4 patients. Capmatinib combined with cetuximab was well tolerated. However, in a phase II study reported by Cathy et al. (NCT01075048), tiantinib in combination with cetuximab did not significantly improve progression-free survival in metastatic CRC (125). This is consistent with the results of tivantinib study in liver cancer carried out by Rimassa et al. (79), indicating that tivantinib has limitations in treating DSTs.

In addition, SU11274 was found to have antitumor effects in CRC treatment by specifically inhibiting c-Met phosphorylation. GAO S H et al. reported that SU11274 can inhibit the proliferation of four colon cancer cell lines (126). At the same time, GAO W et al. also described that SU11274 could induce G1 block and inhibit the survival of CRC cells *in vitro*; what’s more, the growth of xenograft tumors was inhibited *in vivo (* 127). YITAO JIA et al. (120) also studied the effects of c-Met inhibitor PHA665752 on the irradiation activity of colon cancer cells and xenograft cells. The results show that c-Met inhibition makes CRC cells radiation-sensitive by enhancing the formation of DNA double-strand breaks and alleviating tumor hypoxia.

As a c-Met inhibitor, Nororitin (NCTD) is a demethylated analogue of Cantharidin and has strong anti-tumor activity (128, 129). Studies have shown that NCTD can inhibit CRC cell proliferation and induce G2/M growth arrest by reducing the levels of EGFR and c-Met (128). Therefore, NCTD may be also a promising therapeutic agent, and its clinical efficacy and safety are worth looking forward to.

## Application of c-Met monoclonal antibody in DSTs

In recent years, c-Met monoclonal antibody has also been widely used in the treatment of DSTs. The studies mainly include the use of monoclonal antibodies alone, antibodies combination with other drugs and antibody-drug coupling in anti-DSTs (**Table 2**). However, due to its unimproved antitumor activity and high complication rate, it has not been widely applied in clinic.

**Table 2:** Clinical studies on c-Met MAbs in the treatment of DSTs.

Conditions	Interventions	First posted	Number enrolled	Phase	NCT number	State	Status
Neoplasms	Drug:4mg/kg/15mg/kg/30mg/kg Onartuzumab	January 9, 2014	30	Phase 1	NCT02031731	China	Completed
Solid Tumor	Drug: Onartuzumab Drug: Bevacizumab Drug: Erlotinib	July 2, 2015	12	Phase 3	NCT02488330	France	Completed
Colorectal Cancer	Drug: 5-FU Drug:FOLFOX regimen	August 17, 2011	194	Phase 2	NCT01418222	United States	Completed
Gastric Cancer	Drug: 5-Fluoruracil Drug: Folinic acid Drug: Onartuzumab	August 10, 2012	564	Phase 3	NCT01662869	United States	Completed
Hepatocellular Carcinoma	Drug: Onartuzumab Drug: Sorafenib	July 11, 2013	9	Phase 1	NCT01897038	United States	Completed
Gastric Cancer	Drug: Onartuzumab Drug: Oxaliplatin	May 3, 2012	123	Phase 2	NCT01590719	United States	Completed
Solid Cancers	Drug: bevacizumab Drug:MetMAb(PRO143966)	February 17, 2010	44	Phase 1	NCT01068977	United States	Completed
Advanced Cancer Gastric Adenocarcinoma Gastroesophageal Junction Adenocarcinoma	Drug: Emibetuzumab Drug: Ramucirumab	March 10, 2014	97	Phase 1 Phase 2	NCT02082210	United States	Completed
Solid Tumor	Drug: Merestinib	September 30, 2016	12	Phase 2	NCT02920996	United States	Active, not recruiting

All clinicaltrials can be downloaded from www.clinicaltrials.gov (accessed February 28, 2022).

Onartuzumab is a monoclonal antibody that has been evaluated clinically in a variety of human cancers, including targeted therapies alone or in combination (130–132). In 2014, Ravi Salgia et al. (133) evaluated the antitumor activity of onartuzumab in humans for the first time. Thirty-four patients with GC were treated in the study, and only one patient achieved a lasting complete response for nearly two years. In addition, MANISH A. SHAH et al. (134) conducted a Phase II clinical study of onartuzumab combined with oxaliplatin in treating metastatic human EGFR2 negative adenocarcinoma at the gastric or gastroesophageal junction (NCT01590719). The study found that the addition of onartuzumab to c-Met positive GC patients could not improve the outcome. Manish A. Shah, MD et al. (135) also conducted a similar clinical study (NCT01662869) and found that onartuzumab combined with first-line oxaliplatin did not significantly improve clinical prognosis in GC patients with c-Met immunohistochemistry of 2+ and 3+. These studies suggest that onartuzumab has limited efficacy in treating GC.

In addition, the current study found that complications of onartuzumab are also the main reasons limiting its widespread use in clinic. Clinical studies conducted by MANISH A. SHAH et al. found grade 3 adverse events in 88.3% of onartuzumab treated patients, and severe adverse events in 55% (134). Roland Morley et al. (136) conducted a study of complications in 773 solid tumor patients treated with ornatuzumab, all of which were reported possessing edema and venous thromboembolism. Hypoalbuminemia was also more common in the onartuzumab group, with an incidence between 77.8% and 98.3%. Compared with control group, patients treated with onartuzumab had higher rates of arterial venous thromboembolism, gastrointestinal perforation, hypoproteinemia and edema. Therefore, these complications are considered to be expected events of onartuzumab treatment.

Emibetuzumab is a bivalent monoclonal antibody against c-Met that blocks both ligand-dependent and non-ligand-dependent c-Met signaling. S. Betty Yan et al. (137) evaluated emibetuzumab using a c-Met positive GC xenograft model. Emibetuzumab therapy provided transient tumor regression (37.7%), though tumor emerged regrowing during treatment. Daisuke Sakai et al. (138) evaluated the activity and safety of emibetuzumab in the treatment of advanced GC through phase II clinical study. Emibetuzumab was well tolerated in the treatment of advanced GC, but its anti-tumor activity was limited. Later, James J. Harding et al. (139) reported a Phase I/II clinical study of ramucirumab combined with emibetuzumab in the trement of HCC. The results show that the combination of the two antibodies is safe and possesses the activity of inhibiting tumor cells. These studies indicate that emibetuzumab alone has significantly limited antitumor activity and may achieve better efficacy in combination with other antitumor drugs.

Yangbing Jin et al. (140) constructed a new c-Met antibody-drug conjugate, SR-A1403, for the targeted treatment of pancreatic ductal adenocarcinoma with high c-Met expression. Studies have shown that SR-A1403 can significantly inhibit the proliferation, migration and invasion of PC cells, and induce cell cycle arrest and apoptosis. These changes are caused by the inhibition of intracellular cholesterol biosynthesis by SR-A1403. The results illustrate that the SR-A1403-targeting c-Met shows strong preclinical antitumor efficacy in PC. At the same time, Alex Cazes et al. (141) constructed a newly developed antibody drug conjugation, TR1801-ADC, which conjugated a c-Met antibody to a potent pyrrole benzodiazepine toxin conjugation. This study tested TR1801-ADC *in vitro* in PC cell lines and evaluated its preclinical efficacy. Results showed that TR1801-ADC induced a specific cytotoxicity in PC cell lines and produced profound inhibition of tumor growth, even in gemcitabine-resistant tumors. These results reaffirm the importance of c-Met monoclonal antibody in combination with other drugs in treating DSTs.

## The application of adoptive immunotherapy targeting c-Met in DSTs’ treatment

Since the success of CD19-targeting CAR-T in B-cell-derived lymphomas and leukemias, adoptive immunotherapy for solid tumors has been extensively studied. Adoptive immunotherapy has not made a breakthrough in the treatment of solid tumors due to the heterogeneity of solid tumors and inhibitory tumor microenvironment. However, with the rapid development of gene editing technology, it is possible to modify CAR-T cells to adapt to the suppressive tumor microenvironment while enhancing the persistence of tumor killing. Preclinical studies of c-Met-targeting CAR-T in the treatment of DSTs have been conducted mainly in GC and HCC. Two clinical studies of HCC and solid tumor are currently underway in China (NCT03672305 and NCT03638206) in order to evaluate the efficacy and safety of c-Met CAR-T cells and c-Met/PD-L1 CAR -T cells in the treatment of solid tumors.

Nakajima et al. (142) found that c-Met overexpression in GC patients was associated with tumor invasion depth, lymph node metastasis and poor survival rate. Therefore, c-Met has become a potential target for CAR-T cell therapy in GC. Chung Hyo Kang et al. (143) constructed CAR-T cells targeting c-Met, and when incubated with c-Met positive GC cell lines, the secretion of IL-2 and IFN-γ was significantly higher than that of c-Met negative expression ones. Intratumor injection of c-Met CAR-T cells effectively inhibited tumor growth in the xenograft tumor model. In addition, Xingxing Yuan et al. (144) used c-Met-CAR-T cells combined with PD-1 monoclonal antibody in the treatment of GC. By blocking PD-1-PD-L1 binding, novel bifunctional CAR-T cells maintain cytotoxicity to PD-L1+ tumor cells. In tumor tissue, bifunctional CAR-T cells exhibit stronger antitumor ability and prolongation of survival in the PD-L1+ tumor xenograft model compared with c-Met-CAR-T cells. Considering the immunosuppressive microenvironment of solid tumors could inhibit CAR T-cells, our research group constructed c-Met-CAR-T cells for GC and added PD1/CD28 fusion receptor (CSR) to it, aiming to transfer the immunosuppressive signal caused by PD-1 into T cell activation signal (145). PD1/CD28 CSR was found to further enhance the killing capacity of c-Met CAR-T(especially its long-term antitumor effect) and reduce il-6 release levels. CAR-T cells targeting c-Met were found to have no significant off-target toxicity in normal organs. These *in vivo* studies demonstrate antitumor activity by intratumoral injection of c-Met CAR-T cells. On the one hand, local injection in the tumor is not suitable for the later clinical application. On the other hand, it is not conducive to the killing of peripheral circulation and metastatic tumor cells. Therefore, c-Met-CAR-T cells need gene editing to enhance their proliferative activity and persistence.

Wei Jiang et al. (146) constructed bi-specific CAR-T cells targeting c-Met and PD-L1 in the study of HCC. *In vitro* and *in vivo* studies showed that c-Met/PD-L1 CAR-T cells exhibited better antitumor activity against c-Met and PD-L1 positive HCC cells than c-Met CAR T cells or PD-L1 CAR-T cells. These studies also suggest that bi-specific c-Met/PDL1 CAR-T cells are feasible in current T cell engineering techniques. In addition, BING LIU1 et al. (147) constructed c-Met-targeted CAR-NK cells and evaluated their specificity and efficacy for HCC *in vitro*. The results of cytotoxicity assay showed that c-Met CAR-NK cells had stronger specific cytotoxicity against high c-Met expression HCC cell line HepG2. These results suggest that c-Met may be an effective target for adoptive immunotherapy of HCC.

## Discussion

At present, c-Met small molecule inhibitors are widely used in the treatment of DSTs. Most of the preclinical studies showed excellent tumor killing activity, while most patients with c-Met inhibitors alone had limited benefit in clinical studies. Significant improvements in overall survival have been found in several studies when combine c-Met small molecule inhibitors with other antitumor approaches (82, 83, 100, 115, 125). Emibetuzumab was combined with VEGFR-2 monoclonal antibody in treating advanced HCC patients (139). These results may be related to the signal activation mode of c-Met. c-Met could not only bind HGF and then activate downstream signals to promote tumor progression, but also activate downstream oncogenic pathways through molecular interactions with other oncogenic molecules. According to this signal activation mechanism, c-Met can target escape by activating interacting oncogenic molecules (EGFR, RON, etc). Yoshiaki Nakamura et al. (39) demonstrated that cetuximab induces MET gene mutation and amplification in advanced GC. Therefore, the treatment of DSTs should be individualized according to the specific expression of cancer-causing molecules, and multi-target therapy can be combined when necessary. Such a treatment strategy can be as seamless as possible to block the biological signals that promote tumor proliferation and metastasis. It is expected to make breakthrough progress in the treatment of DSTs.

At present, adoptive immunotherapy targeting c-Met is relatively rare in the treatment of digestive system, which is only limited to GC and HCC. Preclinical studies have confirmed its antitumor activity, and some clinical studies are ongoing. According to the c-Met signal activation theory, CAR-T cells targeting c-Met may also target escape in the treatment of DSTs. After all, target escape is one of the major challenges adoptive immunotherapy faces in treating solid tumors. Therefore, according to c-Met-CAR T cells and its interacting molecules, targeted therapies may achieve better clinical efficacy.

Furthermore, complications also limit the widespread clinical use of small-molecule inhibitors and monoclonal antibodies targeting c-Met. The study of tivantinib in metastatic HCC found 10 neutropenia and 8 anemia in 48 patients in the tivantinib group (78). Phase I adverse events related to SAR125844 in GC were also common in these patients (112). Onartuzumab was found to have serious adverse events in 55% of patients with GC, with edema and venous thromboembolism reported in all treated patients (134, 136). Therefore, it is necessary to investigate the risk factors for these complications, so as to pave the way for the application of c-Met targeted therapy in DSTs.

## Conclusion

In conclusion, existing preclinical trials have demonstrated the potential of targeting c-Met for DSTs. The majority of clinical studies using targeted c-Met therapy alone have failed to achieve better efficacy in treating DSTs, while the combination of targeted c-Met therapy with other antitumor methods demonstrates its great potential. According to the biological function of c-Met in promoting tumor progression and inducing drug resistance, targeted therapy with c-Met and its associated oncogenic molecules should be explored to achieve better efficacy It is believed that with the optimization of c-Met targeted therapy, the treatment of DSTs will make a breakthrough.

## Author contributions

All authors conceptualized and wrote the manuscript. ZZ and DL additionally performed literature and data analysis. All authors contributed to the article and approved the submitted version.

## Funding

This study was funded by the Third Affiliated Hospital of Gansu University of Chinese Medicine (Project Approval Number: 2016YG-06) and Baiyin City 2019 Science and technology plan project (Project Approval Number: 2019-1-22Y).

## Acknowledgments

Thanks to all the authors who participated in the design and data analysis of this paper, as well as the Third Affiliated Hospital of Gansu University of Traditional Chinese Medicine for providing convenience.

## Conflict of interest

The authors declare that the research was conducted in the absence of any commercial or financial relationships that could be construed as a potential conflict of interest.

## Publisher’s note

All claims expressed in this article are solely those of the authors and do not necessarily represent those of their affiliated organizations, or those of the publisher, the editors and the reviewers. Any product that may be evaluated in this article, or claim that may be made by its manufacturer, is not guaranteed or endorsed by the publisher.
